# Risk assessment scales to predict risk of lower extremity deep vein thrombosis among multiple trauma patients: a prospective cohort study

**DOI:** 10.1186/s12873-023-00914-7

**Published:** 2023-12-05

**Authors:** Huijuan Chen, Libing Sun, Xiangyan Kong

**Affiliations:** 1 Nursing department, Peking University People’s Hospital, No.11 Xizhimen South St. Xicheng District, Beijing, 100044 China; 2 Trauma center, Peking University People’s Hospital, National Center for Trauma Medicine, No.11 Xizhimen South Street, Xicheng District, Beijing, 100044 China

**Keywords:** Deep vein thrombosis, Multiple trauma, Prediction, Prophylaxis, Risk assessment model

## Abstract

**Background:**

Deep vein thrombosis (DVT) is a common complication in orthopedic patients. Previous studies have focused on major orthopedic surgery.There are few studies with multiple trauma. We aimed to describe the prevalence of DVT and compare the predictive power of the different risk assessment scales in patients with multiple trauma.

**Methods:**

This prospective cohort study involved multiple trauma patients admitted to our hospital between October 2021 and December 2022. Data were prospectively collected for thrombotic risk assessments using the Risk Assessment Profile for thromboembolism(RAPT), the DVT risk assessment score (DRAS), and the Trauma Embolic Scoring System (TESS), respectively. The receiver operation characteristic (ROC) curve and the area under the curve (AUC) were evaluated to compare the predictive power. The whole leg duplex ultrasound of both lower extremities Doppler ultrasound was used to determine DVT incidence.

**Results:**

A total of 210 patients were included, and the incidence of DVT was 26.19%. Distal DVT accounted for 87.27%; postoperative DVT, 72.73%; and bilateral lower extremity thrombosis, 30.91%. There were significant differences in age, education degree, pelvic fracture, surgery, ISS, D-dimer level, length of hospital stay and ICU stay between the thrombosis group and the non-thrombosis group. The AUCs for RAPT, DRAS, and TESS were 0.737, 0.710, and 0.683, respectively. There were no significant differences between the three ROC curves.

**Conclusions:**

The incidence of DVT was relatively high during hospitalization. We prospectively validated the tests to predict risk of DVT among patients with multiple trauma to help trauma surgeons in the clinical administration of DVT prophylaxis.

## Introduction

Around the world, trauma is responsible for 5.7 million deaths annually, accounting for 25–33% of unintentional deaths of those under 45 years of age and 90% of the global trauma burden in low- and middle-income countries [1]. With the rapid development of China’s modernization, multiple, critical, and mass trauma caused by high-energy hazards have been on the rise [2]. Multiple trauma, also known as polytrauma, is an injury to two or more anatomical sites caused by a single factor, of which at least one site is life-threatening. The interaction between injury regions with multiple injuries can aggravate the condition, the clinical treatment is difficult, and the patients who survived the trauma are at risk of life-threatening complications, such as respiratory complications, multiple organ dysfunction syndrome, and/or venous thromboembolism (VTE)) [3–5].

VTE including DVT and pulmonary embolism (PE), is a potentially preventable and important medical problem that increases morbidity, mortality, disability, and medical costs in hospitalized patients [3, 6]. The incidence of VTE in patients with multi-system or major trauma is reported to be between 4 and 60% [7, 8]. Previous studies have mainly focused on major orthopedic surgery, especially hip fractures surgery [9], and there are few studies on DVT risk assessment in patients with multiple trauma. Patients with multiple injuries are at higher risk of DVT. Apart from common orthopedic risk factors, it may be related to trauma-induced coagulopathy, increased risk of bleeding, delayed thromboprophylaxis (e.g., patients with active bleeding, coagulopathy, hemodynamic instability, solid organ injury, traumatic brain injury, or spinal cord injury), and prolonged immobilization from being trapped at the trauma site or in the hospital [10–14]. While treating patients with multiple injuries, early DVT risk assessment and a targeted preventive strategy is critical for the success rate and health outcomes of patients.

Color Doppler ultrasound (DUS) is currently the most widely used examination method for the clinical diagnosis of VTE [15], and has the characteristics of safety, non-invasiveness, timeliness, and reproducibility. The use of ultrasound screening for VTE in trauma patients is still controversial, there are no data or guidelines to support routine screening, and more and more scholars advocate that high-risk or symptomatic patients should be screened according to reasonable assessments as needed [16]. Multiple risk assessment models were developed to improve risk stratification of patients and guide prophylaxis management. Due to the peculiarity of multiple trauma, surgeons may be unable to obtain more comprehensive variables quickly and accurately, resulting in some prediction models that may not be suitable for DVT in patients with multiple injuries.

In 1997, Greenfield et al. [17] proposed the RAPT to predict the risk of DVT in trauma patients. The score screens traumatic patients at risk by underlying condition, iatrogenic factors, injury-related factors, and age [18]. The Guideline recommended that RAPT be evaluated at all hospitalized orthopedic trauma patients [19]. The TESS was proposed by Rogers et al. in 2012 [20], and has been proven to predict the risk of VTE in the trauma population. TESS is determined from five clinical variables: age,the Injury Severity Score (ISS),body mass index (BMI), ventilator days, and presence of a lower extremity fracture. Studies have shown that the area under the ROC curve was 0.89, with a sensitivity of 81.6% and specificity of 84% [20]. Peng et al. [21] believe that, for trauma patients, a risk prediction model for early identification of DVT should be established based on routine electronic clinical data. In 2020, they developed and prospectively validated the DRAS for multiple trauma patients based on the demographic information of patients and routine clinical data easily obtained within a few hours after admission in a level I trauma center [21]. And the area under the ROC curve of the prediction model was 0.890 (0.841–0.940), with a sensitivity of 88.90%, and specificity of 77.70%.The RAPT and TESS models were developed specifically for trauma patients, and DRAS specifically for multiple trauma; however, the effect of the above scale on the risk assessment of DVT in multiple trauma patients is unclear. We aimed to select a perioperative DVT risk assessment tool suitable for patients with multiple injuries to guide medical staff in early intervention and ensure the safety of high-risk patients.

## Patients and methods

### Study design

We carried out a prospective cohort study of patients presenting with multiple trauma admitted to a trauma center between October 2021 and December 2022. We recruited all patients admitted with a diagnosis of multiple trauma to our trauma center ward through our emergency department. The inclusion criteria were as follows: 1) age ≥ 18 years; 2) color DUS performed at least twice (at admission and during hospitalization), and the first test was negative; 3) no cognitive, verbal, or intellectual impairment; 4) complete demographic and clinical information; and 5) voluntary participation in this study. The exclusion criteria were as follows: 1) a preexisting thrombotic disease with ongoing treatment; or 2) family history of inherited thrombophilic defects. This study was approved by the ethics committee of Peking University People’s Hospital (2021PHB294-001).

The multiple trauma patients were screened according to the inclusion and exclusion criteria within 24 h after admission. After the patient signed an informed consent form for the study, the assessors used three assessment tools to assess the DVT risk of the patients who met the inclusion criteria:1) RAPT; the score screens traumatic patients at risk by underlying condition, iatrogenic factors, injury-related factors, and age. A score ≤ 5 is considered as low risk; 5–14, moderate-risk; ≥ 15, high risk. 2) DRAS; calculated based on available data for age, BMI, presence of a lower extremity fracture, ISS, D-dimer, fibrinogen degradation products (FDPs) level, and prothrombin time (PT). According to the score, < 132 is low risk; 132–208 is medium risk; 209–278 is high risk: and > 278 is very high risk. 3) TESS; determined from five clinical variables: age, ISS, BMI, ventilator days, and presence of a lower extremity fracture. A TESS score of 0–2 is not considered to be at risk for VTE; 3–6, low risk; and 7–14, moderate to high risk. The trauma severity of patients was assessed by an independent clinician using ISS. All patients were evaluated by the same investigator.

Basic data were collected prospectively from electronic medical records. When the investigator had doubts about the records of the electronic medical record system, the details were confirmed with the patient or the doctor in charge. The first assessment was completed within the first 24 h after admission, the dynamic assessment was performed when there were changes in risk factors, and the data was analyzed with the worst value of each patient.

### Variables

The baseline data and clinical characteristics of all patients were reviewed. We collected the following demographic information from the enrolled patients: age, sex, BMI, injury type, smoking, education degree, length of hospital stay, length of ICU stay, and current complications (hypertension, diabetes) If diabetes was present, we determined whether the blood glucose level was > 10 mmol/L. We also included information on disease and treatment, including mechanism of injury, ISS, area of injury, time from injury to admission, chemoprophylaxis, and mechanical prevention. Laboratory variables were collected based on routine blood examinations, including D-dimer level.

### Outcomes

We monitored whether a patient was diagnosed with DVT through the daily electronic medical record system daily, and recorded the examination time of the lower extremity venous Doppler exam and the DVT location and type. The study endpoint was the occurrence of DVT during hospitalization or patient discharge. All data were recorded using Epidata 4.2.0.

### Statistical analysis

Statistical analysis was performed using SPSS 23.0 (SPSS Inc, Chicago, IL,USA). Data were presented as mean ± SD or median with inter-quartile ranges for continuous variables and frequencies with percentages for categorical variables. The T-test or the Mann–Whitney U test was used for comparison of continuous variables between the groups. Normality test was applied. The Chi square test was used for comparison of categorical variables between the groups. Using the risk scores as the independent variables and the diagnosed DVT results as the dependent variable, the receiver operating characteristic (ROC) curves and the areas under the ROC curve (AUCs) were compared. The greater the AUC, the higher the diagnostic accuracy. AUCs of 0.5–0.7, 0.7–0.9, and > 0.9 indicated poor, moderate, and excellent diagnostic accuracy, respectively. Sensitivity and specificity were compared at the best dividing point of the three scales. The *P* values, odds ratios, and 95% confidence intervals (CIs) of the selected features were assessed. *P* value < 0.05 was considered as statistically significant.

## Results

### Patient demographics

Two hundred and ten patients (149 males and 61 females) met the inclusion criteria and were included in the study. Fifty-five patients were screened by lower extremity DUS. The incidence of DVT was 26.19%0.4 out of 55 DVT patients had symptomatic DVT, and the incidence of symptomatic DVT was 7.27% (4/55). The demographic data are shown in Table 1.

**Table 1:** Comparison of the potential risk factors between the thrombosis and non-thrombosis patients (*n* = 210)

Factors	All patients (*n* = 210)	Thrombosis group (*n* = 55)	Non-thrombosis group (*n* = 155)	Statistics	*P* value
Sex					
Male	149 (71%)	35 (63.64%)	114 (73.55%)	1.935	0.164
Female	61 (29%)	20 (36.36%)	41 (26.45%)		
Education degree					
Primary school and below	57 (27.14%)	24 (43.64%)	33 (21.29%)	11.686	**0.009**
Junior middle school	94 (44.76%)	18 (32.73%)	76 (49.03%)		
high school	37 (17.62%)	10 (18.18%)	27 (17.42%)		
Junior college and above	22 (10.48%)	3 (5.45%)	19 (12.26%)		
Smoking					
Yes	82 (39.05%)	22 (40%)	60 (38.71%)	0.028	0.866
No	128 (60.95%)	33 (60%)	95 (61.29%)		
High blood pressure					
Yes	50 (23.81%)	18 (32.73%)	32 (20.65%)	3.267	0.071
No	160 (76.19%)	37 (67.27%)	123 (79.35%)		
High blood glucose (> 10 mmol/L)					
Yes	13 (23.81%)	4 (23.81%)	9 (23.81%)	0.747	0.457
No	197 (23.81%)	51 (23.81%)	146 (23.81%)		
Age^a^	49.12 ± 15.91	57.24 ± 12.54	46.24 ± 16.01	5.176	**0.000**
BMI^a^	24.29 ± 3.79	24.61 ± 3.85	24.18 ± 3.78	0.722	0.471

^a^Independent sample t-test

### Diseases and treatment

Most patients with multiple trauma were injured by road traffic injury (61.91%). As for ISS, there were 80 cases with an ISS < 16, 88 cases with 16 ≤ ISS < 25, and 42 cases with ISS ≥ 25. The median ISS was 17 (13,22). The incidence of DVT in patients with ISS ≥ 16 was 30.77% (40/130) and the incidence in those with ISS < 16 was 18.75% (15/80). The data on diseases and treatments of all patients are presented in Table 2.

**Table 2:** Diseases and treatments of all patients (*n* = 210)

Factors	All patients (*n* = 210)	Thrombosis group (*n* = 55)	Non-thrombosis group (*n* = 155)	Statistics	*P* value
Mechanism of injury					
Road traffic injury	130 (61.91%)	34 (61.82%)	96 (61.94%)	0.479	0.787
Fall	65 (30.95%)	16 (29.09%)	49 (31.61%)		
Other injury^a^	15 (7.14%)	5 (9.09%)	10 (6.45%)		
Area of injury					
Head	96 (45.71%)	25 (45.45%)	71 (45.81%)	0.002	0.964
Chest	150 (71.43%)	38 (69.69%)	112 (71.79%)	0.200	0.655
Abdomen	55 (26.19%)	18 (32.73%)	37 (23.87%)	1.647	0.199
Spine and spinal cord	73 (34.76%)	21 (38.18%)	52 (33.55%)	0.384	0.535
Pelvis	60 (28.57%)	23 (41.82%)	37 (23.87%)	6.407	**0.011**
Extremities	122 (58.10%)	33 (60%)	89 (57.42%)	0.111	0.739
Number of operations					
0	49 (23.33%)	9 (16.36%)	40 (25.81%)	7.365	**0.025**
1	121 (57.62%)	29 (52.73%)	92 (59.35%)		
≥ 2	40 (19.05%)	17 (30.91%)	23 (14.84%)		
Chemoprophylaxis	134 (63.81%)	47 (85.45%)	87 (56.13%)	15.118	**0.000**
Time from injury to admission* (h)^b^	9 (6,13.25)	8 (6,14)	9 (5,13)	-0.406	0.684
Injury Severity Score^b^	17 (13,22)	22 (15,27)	17 (13,22)	-3.154	**0.002**
Length of hospital* (days)^b^	16 (11,23)	21 (15,29)	14 (9,21)	-4.577	**0.000**
Length of ICU* (days)^b^	0 (0,7)	1 (0,15)	0 (0,5)	-2.286	**0.022**
D-dimer (mg/L, M [Q1, Q3])	3.23 (1.60,6.70)	5.77 (3.27,9.82)	2.63 (1.08,5.01)	-5.171	**0.000**

^a^Included bruise injury by heavy object, gas explosion, violent attacks, stab wounds, power saw injury

^b^Mann-Whitney U test. Others were tested using the Chi square test

### Prevalence of DVT

In the DVT group, 48/55 (87.27%) multiple trauma patients had distal DVT, 5/55 (9.09%) had proximal DVT, and 2/55 (3.64%) had mixed DVT. The prevalence of DVT was 30.91% (17/55), 38.18% (21/55), and 30.91% (17/55) in the left, right, and both lower limbs, respectively. In total, 72.73% occurred after surgery. The median time from acute trauma to DVT was 8 (5,12) days. The DVT distribution of the 55 patients is shown in Table 3.

**Table 3:** DVT distribution of 55 patients

	**Category**	**Number (%)**
Thrombosis location	Distal DVT	48 (87.27)
	Proximal DVT	5 (9.09)
	Mixed DVT	2 (3.64)
Time of occurrence	Before operation	15 (27.27)
	Post operation	40 (72.73)
Limb of thrombosis	Left	17 (30.91)
	Right	21 (38.18)
	Left + right	17 (30.91)

### Incidence of DVT in different risk stratifications

For all patients, the DVT risk levels are shown in Table 5 and compared by the Chi square test. The incidence of DVT increased significantly with risk level and the difference was statistically significant (*P* < 0.001). In the RAPT assessment, 23 (41.82%) multiple trauma patients were classified into the low-risk group, with 4.35% developing DVT; 152 (72.38%) were classified into the medium-risk group, with 22.37% developing DVT; and 35 (16.67%) were classified into the high-risk group, with 57.14% developing DVT. In the DRAS assessment, 56 (26.67%) multiple trauma patients were classified into the low-risk group, with 10.71% developing DVT; 82 (39.05%) into the medium-risk group, with 23.17% developing DVT; 30 (14.29%) into the high-risk group, with 30.0% developing DVT; and 42 (20.0%) were classified into the very high-risk group, with 50.0% developing DVT. In the TESS assessment, the results suggested that 21 (10%) were divided into the no-risk group, with 9.52% developing DVT; 118 (56.19%) into the low-risk group, with 18.64% developing DVT; and 71 (33.81%) into the high-risk group, with 43.66% developing DVT (Table 4).

**Table 4:** Associations of different risk groups based on RAPT, DRAS, and TESS score with incidence of DVT

DVT risk classification	RAPT		DRAS		TESS	
	**Trauma**	**DVT**	**Trauma**	**DVT**	**Trauma**	**DVT**
None	-	-	-	-	21	2 (9.52)
Low	23	1 (4.35)	56	6 (10.71)	118	22 (18.64)
Medium	152	34 (22.37)	82	19 (23.17)	-	-
High	35	20 (57.14)	30	9 (30.00)	71	31 (43.66)
Very high	-	-	42	21 (50.00)	-	-
^χ2^		24.171		19.867		17.705
P		0.000		0.000		0.000

### ROC and AUC results

**Fig. 1:**
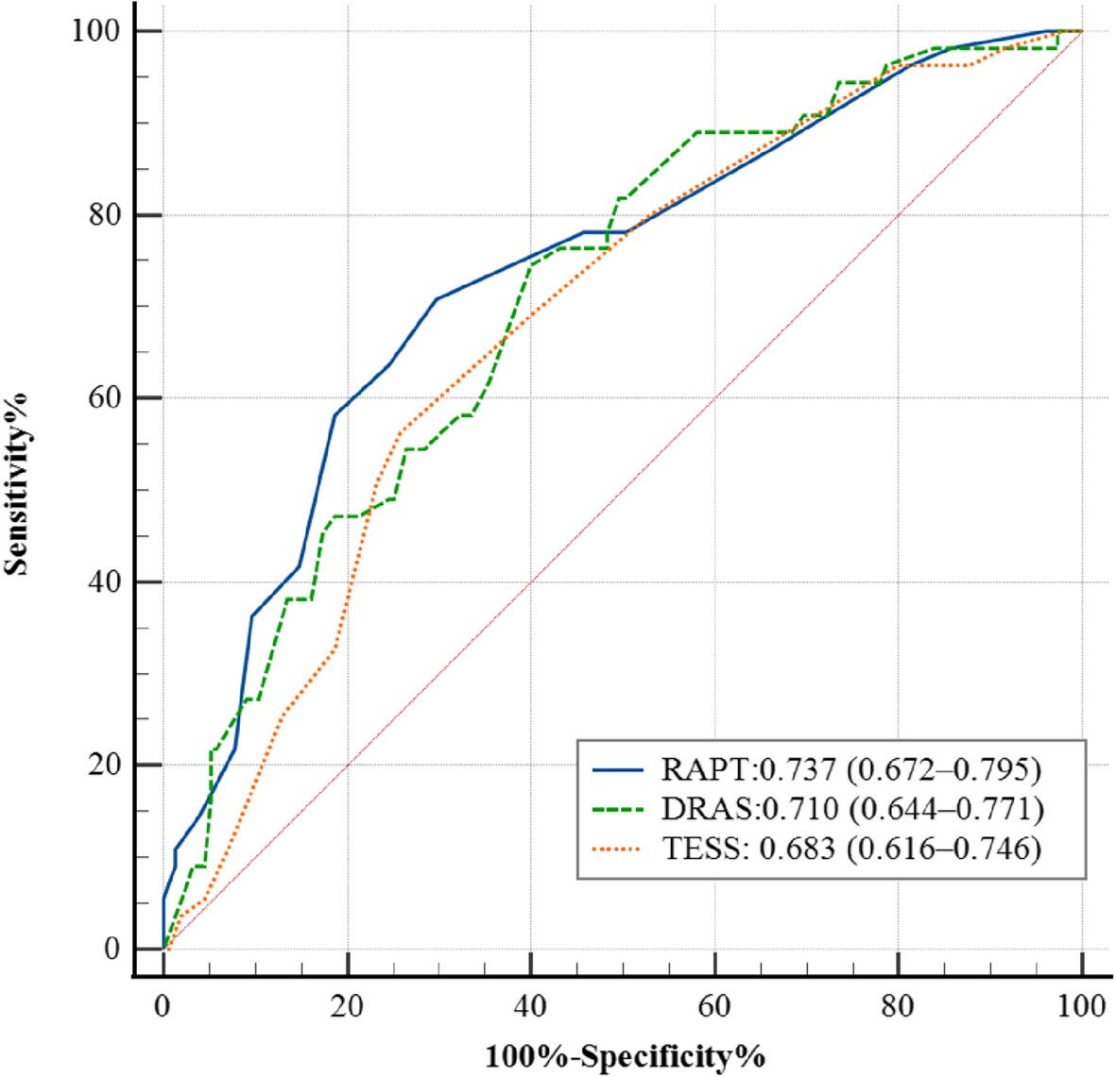
ROC analysis of the RAPT, RAPT, and TESS scores

According to the sensitivity and specificity of the DVT test results, the diagnostic value of the statistical evaluation tool was determined by ROC analysis. In the screened group, the ROC curve was drawn, and the AUC was calculated according to the outcome of DVT. The ROC curve analysis showed that the AUC of RAPT was 0.737 (0.672–0.795), with 70.9% sensitivity and 70.3% specificity; DRAS, 0.710 (0.644–0.771), with 74.6% sensitivity and 60.0% specificity; and TESS, 0.683 (0.616–0.746), with 56.4% sensitivity and 74.2% specificity. The results demonstrated that the RAPT had better predictive value (Tables 5 and 6) (Fig. 1).

**Table 5:** AUC analysis of the RAPT, RAPT, and TESS scores

Variable	AUC	Standard error	Significance	95% CI	
				**Lower limit**	**Upper limit**
RAPT	0.737	0.0396	< 0.001	0.672	0.795
DRAS	0.710	0.0392	< 0.001	0.644	0.771
TESS	0.683	0.0396	< 0.001	0.616	0.746

**Table 6:** Area under the ROC curve of the RAPT, DRAS, and TESS

Variable	Z value	*P* value
RAPT and DRAS	0.664	0.507
DRAS and TESS	0.677	0.498
RAPT and TESS	1.357	0.175

## Discussion

Two hundred and ten patients were included in the study, and the incidence of DVT was 26.19%. Among the DVT patients, distal DVT accounted for 87.27%, postoperative DVT for 72.73%, and bilateral lower extremity thrombosis for 30.91%. There were significant differences in age, education degree, pelvic fracture, operation, ISS, D-dimer level, length of hospital, and length of ICU stay between the thrombosis group and the non-thrombosis group. The AUCs for RAPT, DRAS, TESS were 0.737, 0.710, and 0.683, respectively. RAPT had better predictive value, but there were no significant differences between the three ROC curves.

Previous studies investigated the incidence and risk factors of DVT in orthopedics patients, but few assessed the prevalence of DVT in multiple trauma population groups. In this study, 55 patients were diagnosed with DVT, accounting for 26.19%, and distal DVTs were dominant (87.27%). In a study of 716 trauma patients, adequate venography found that 58% of patients had lower extremity DVT; 18% had proximal DVT [22]. However, the incidence of lower extremity DVT in trauma patients who have received thromboprophylaxis was still as high as 12 ~ 65% [23]. Sun et al. [24] found that, even with conventional prevention, the incidence of DVT in multiple trauma patients was as high as 42.08%, of which the incidence of proximal DVT was 6.56% and that of distal DVT was 35.52%. The DVT prevalence in this study was similar to others. Of course, this study was a prospective observational study and we did not change the conventional routine of clinical DVT prevention. In our study, 85.45% of patients in the thrombus group received thromboprophylaxis, which was statistically significant compared with the control group (56.13%). Since 2017, our hospital has standardized and established an in-hospital VTE Prevention and Treatment Expert Group and compiled the in-hospital VTE Prevention and Treatment Manual. In the same year, the Nursing Department established a nursing working group for departments with high VTE risks and gradually developed a multidisciplinary and standardized basic thromboprophylaxis, mechanical thromboprophylaxis, and chemical thromboprophylaxis program. This may have some impact on the data of this study.

We also found that the preoperative DVT incidence was 27.27%. Therefore, DVT risk assessment should be carried out for multiple trauma patients before surgery, as well as DUS diagnostic testing for high-risk groups to reduce the economic burden of conventional ultrasound screening [25]. At the same time, the size and location of the embolus could be assessed by ultrasound, and an inferior vena cava filter could be placed, if necessary, to ensure the safety of the surgery. Although the incidence of unilateral lower extremity DVT in this study was high and mainly occurred in the injured limb, it was still necessary to be alert to the possibility of DVT in the healthy limb. In our study, 17 (30.91%) patients developed bilateral DVT, and none of the patients had bilateral fractures.

The data of this study showed that the difference between the thrombotic group and the non-thrombotic group in age, education, pelvic fractures, surgery, ISS, and D-dimer levels were statistically significant. In addition, the number of days in the hospital and the number of days in the ICU in the thrombus group were significantly longer, and the difference was statistically significant. Length of hospital stay, age, major trauma or fracture, major surgery, and D-dimer are common and significant predictors of DVT in patients [9, 26, 27]. Liasidis et al. [28] pointed out that, despite mechanical and chemical thromboprophylaxis, the risk of DVT in patients with pelvic fractures is still very high, which might be related to long-term immobilization. In addition, the higher the education level of patients, the lower the incidence of DVT, which may be related to the higher acceptance of the disease, thromboprophylaxis knowledge, and the higher compliance with thromboprophylaxis practice. ISS, as the most commonly used assessment tool for the disease severity of multiple trauma patients, was considered to be closely related to the risk stratification of DVT in previous studies [29–31]. We also confirmed that the ISS of the thrombotic group was significantly higher than that of the non-thrombotic group, and the difference was statistically significant.

Validated risk assessment tools contribute to better risk classification and guide prophylaxis. Table 6 demonstrated that the RAPT had better predictive value among the three scales, the AUC was 0.737 (0.672–0.795) with 70.9% sensitivity and 70.3% specificity, but the difference was not statistically significant. A prospective study of 2,281 trauma patients showed that RAPT score was a good predictor of VTE and, in moderate-risk and high-risk patients, the RAPT had a sensitivity of 0.82, a specificity of 0.57, and the AUC was 0.72 [18]. In recent years, an increasing number of studies have supported the use of routine clinical characteristics and laboratory tests to aid decision making [32]. Peng et al. [21] developed a novel risk score for multiple trauma based on patient demographic information and routine clinical variables that can be obtained easily within a few hours after admission. The DRAS was constructed by combining the seven predictors, including age, BMI, lower extremity fracture, ISS, D-dimer, FDPs, and PT. The items of DRAS score were objective and realistic, and simple to operate. The AUC of the DRAS score in this study was 0.710 (0.644–0.771), with 74.6% sensitivity and 60.0% specificity. The sensitivity was highest among the three scales in this study, but the specificity was the lowest. This may be related to the limited sample size of this study, which needs to be further explored.

The TESS score is also one of the commonly used scales for DVT risk identification in trauma patients. We performed this analysis to assess the predictive ability of the TESS score in the same study cohort, and the results demonstrated that the AUC of TESS was 0.683 (0.616–0.746), with 56.4% sensitivity and 74.2% specificity. The RAPT and DRAS had a better predictive value when compared with the TESS score. There might be two reasons for the difference. First, the TESS includes variables associated with venous stasis (age, obesity, ventilator days) and vascular endothelial injury (lower extremity fractures), but fails to include variables related to hypercoagulability and others, such as D-dimer and tranexamic acid(TXA) administration [33, 34]. Second, an optimal high-risk cut-off value of ≥ 7 in TESS demonstrates high sensitivity in predicting VTE. We found an incidence of 18.64% in low-risk patients. TESS, therefore, fails to correctly stratify a clinically significant number of patients, but still has the advantage of simplicity in calculation as it contains only five clinical variables. TESS is also less cumbersome than RAPT, which has also been shown to model VTE risk in trauma patients but contains 15 clinical variables [34]. Because of the urgent nature of trauma care, some indicators in the above models may not be available 24 or 48 h after admission. Furthermore, a simple, practical, quick, and effective prediction method should be recommended for any trauma surgeon in consideration of the specialty of acute trauma.

There were some limitations in this study. First, the sample size was limited. Although we included all patients who met the inclusion criteria during the study period, the sample size was only 210 cases, due to limited observation time and the impact of COVID-19. Therefore, the analyses may have been underpowered for the detection of significant differences in some outcome parameters. In addition, there was only one patient diagnosed of PE during our study period, and thus we did not include PE as an outcome variable. Moreover, as this was a single trauma center study, the external validity and generalizability of the results in other hospital settings are unknown.

## Conclusions

Major trauma is a potent precipitating factor of DVT. During the early treatment of multiple trauma patients, DVT risk assessment and targeted prevention is critical to the treatment success rate and health outcomes. In our study, there were no significant differences between the three ROC curves, but considering the clinical significance of their trends, the RAPT was more likely to identify DVT patients with multiple trauma, followed by DRAS. And additional data are needed to confirm this conclusion in future studies. However, both of the models also have their restrictions. In addition, this study also demonstrated that the incidence of DVT in multiple trauma patients was relatively high, which requires the attention of the healthcare professional to recognize high DVT risk patients and prescribe suitable thromboprophylaxis. Due to the peculiarity of multiple trauma and the urgency of treatment, further research should focus on developing an early, convenient, and sensitive DVT assessment tool which can be evaluated by capturing routine data from the clinical patient electronic medical record system.

## Data Availability

The data that support the findings of this study are available from the corresponding author upon reasonable request.
